# Supplementary material for: Association between Antidepressant Treatment during Pregnancy and Postpartum Self-Harm Ideation in Women with Psychiatric Disorders: A Cross-Sectional, Multinational Study
Source: Int J Environ Res Public Health. 2020 Dec 23;18(1):46. doi: 10.3390/ijerph18010046 (PMC7793536; doi:10.3390/ijerph18010046)
Supplement: Supplementary file 1 [file ijerph-18-00046-s001.pdf]

SUPPLEMENTARY TEXT

METHODS

Additional details on "Postpartum self-harm ideation"

Validated translated versions of the original EPDS were available for eight languages other than English.[1] For the Serbian version, translation and back-translations were carried out by two independent linguistic experts and any discrepancies between the back-translated and original EPDS were settled. For the remaining five languages, we utilized translated versions used in previous studies.[2-5] The EPDS developers were acknowledged in each electronic questionnaire under the section presenting the scale.

Additional details on "Data analysis"

The statistical office of the European Union provided information about the distribution of these variables among women of childbearing age in each participating country, except Russia.[6] For the latter, we used average data for the other Eastern European countries.[6,7] For USA, Australia and Canada, we used relevant national sources.[8-10]

The propensity score was estimated using the survey weight and the following maternal covariates: having previous children, marital status, region of residency, occupation at time of conception, educational attainment and age, immigrant status, smoking and alcohol use during pregnancy, perinatal use of folic acid, maternal neurotic traits, sleeping problems in pregnancy, contact with healthcare providers due to fertility problems, and use of other medications in pregnancy, that is benzodiazepines and z-hypnotics, and antipsychotics. These covariates were selected on the basis of subject knowledge, prior research, and characteristics of the resulting propensity score.

Power analysis

The study was adequately powered to detect a 60% reduced risk in self-harm ideation, as antidepressant treatment was expected to lower the frequency of this outcome, with α set to 0.05 and 80% power. Because of risk of self-harm was greater with antidepressant exposure, posthoc power analysis showed that we could rule out a risk as large as 1.85, but not a smaller effect size.

Sensitivity analyses

To examine the robustness of our results, we conducted a set of sensitivity analyses. First, we replicated the main analyses after inclusion of 14 women in the early postpartum period (0–4 weeks after birth). Then, we excluded five women who reported psychiatric disorders other than depression or anxiety. Because women were enrolled in the study at different timings since birth, we tested the statistical significance of the interaction term between antidepressant treatment and child age at the time of questionnaire completion. Because the IPTW weighting could not fully minimize differences in some baseline between the exposure groups (i.e., use of benzodiazepines and z-hypnotics in pregnancy, maternal age, contact with healthcare due to infertility), we additionally adjusted the fully weighted outcome models for these variables.

RESULTS

Additional details on "Results"

The composite IPTW had a mean of 1.00 (sd: 1.14) for antidepressant ever in pregnancy exposure, with range 0.10-9.19. For antidepressant in three or one trimesters, the IPTW means were respectively 0.98 (sd: 1.06) and 0.99 (sd: 1.31), with ranges 0.11-7.27 and 0.10-12.75.

Sensitivity analysis

As reported in Supplementary Table 2, inclusion of women in the earliest postnatal period (0-4 weeks since childbirth, n=14, 4 medicated with antidepressants during pregnancy and 10 non-medicated) did not meaningfully affect the association measures. We did not observed any statistical significant interaction between antidepressant in pregnancy and child age at the time of questionnaire completion. Similarly, we found no interaction between antidepressant treatment ever in pregnancy and maternal age in the age band 17-24, 25-30 and over 30 years (p-values for interaction terms were respectively 0.199 and 0.687). Accounting for the random effect by country of residency did not materially change the magnitude of the association measures of the main analysis, but the 95% CI of the point estimate for antidepressant ever in pregnancy and SHI became narrower and borderline significant (p=0.080). The other associations remained materially unchanged.

REFERENCES

1. Cox JL, Holden J. *Perinatal mental health: a guide to the Edinburgh Postnatal Depression Scale (EPDS).* RCPsych Publications; 2003.
2. Department of Health. Edinburgh Postnatal Depression Scale (EPDS): Translated versions – validated. Perth, Western Australia: State Perinatal Mental Health Reference Group. In:2006.
3. National Institute for Health and Welfare. http://www.thl.fi/thl-client/pdfs/760aa5ca-d9bd-4279-9d17-b1e8a58d00e8. Accessed May 13, 2011.
4. Grote V, Vik T, von Kries R, et al. Maternal postnatal depression and child growth: a European cohort study. *BMC Pediatr.* 2010;10:14.
5. Stewart DE, Gagnon A, Saucier JF, Wahoush O, Dougherty G. Postpartum depression symptoms in newcomers. *Can J Psychiatry.* 2008;53(2):121-124.
6. Eurostat. Population by educational attainment level, sex and age (%) - main indicators. http://ec.europa.eu/eurostat/web/products-datasets/-/edat_lfse_03. Published 2012. Accessed 14 AUgust, 2017.
7. Applied Survey Methods - A statistical perspective. http://applied-survey-methods.com/weight.html. Published 2017. Accessed 14 August 2017.
8. Hamilton B, Martin J, Ventura S. *National Vital Statistics Reports. Births: Preliminary Data for 2010.* U.S. Department of health and human services. Centers for Disease Control and Prevention. National Center for Health Statistics. National Vital Statistics System;2011.
9. Statistics Canada. Births. http://www.statcan.gc.ca/daily-quotidien/111220/dq111220g-eng.htm. Published 2010. Accessed 12 November 2012.
10. Australian Bureau of Statistics. Births, Australia. http://www.abs.gov.au/AUSSTATS/abs@.nsf/mf/3301.0. Published 2011. Accessed November 13th, 2012.

SUPPLEMENTARY FIGURE

Figure S1: Crude proportion of frequency of self-harm ideation by antidepressant use among 187 women with depression/anxiety during pregnancy

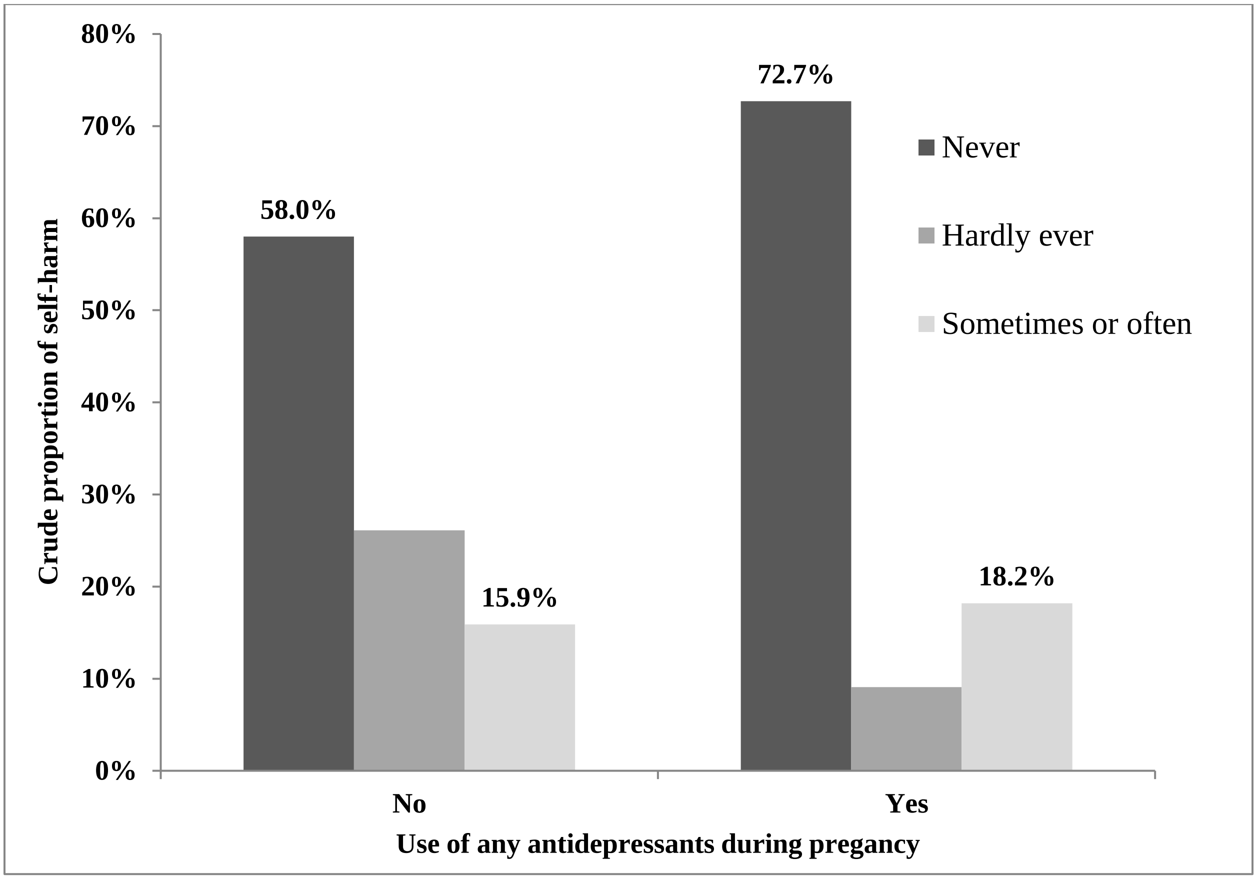

SUPPLEMENTARY TABLES

Table S1: Standardized mean differences of baseline covariates before and after weighting by the inverse probability of treatment weighting for antidepressant exposed versus unexposed.

	**Before weighting**	**After weighting**	**Before weighting**	**After weighting**	**Before weighting**	**After weighting**
	Antidepressant, ever in pregnancy vs non-exposure		Antidepressant for 3 trimesters vs non-exposure		Antidepressant for 1 trimester vs non-exposure	
Characteristics	SMD		SMD		SMD	
Neuroticism	-0.166	0.122	-0.155	0.124	-0.165	0.000
Previous children	0.040	0.027	0.091	0.031	0.000	0.233
Benzodiazepine and z-hypnotic use during pregnancy	0.228	0.313	0.147	0.147	0.336	0.235
Marital status	-0.172	-0.018	-0.259	-0.133	-0.063	0.598
Alcohol use after awareness of pregnancy	-0.215	-0.052	-0.214	-0.041	-0.565	-0.390
Smoking during pregnancy	-0.109	-0.061	0.124	-0.082	-0.123	0.426
Northern Europe	0.389	0.161	0.462	0.277	0.211	0.247
Eastern Europe	-0.772	-0.363	-0.885	-0.479	-0.439	-0.290
North America	-0.041	0.093	0.023	0.070	-0.251	-0.233
Australia	0.189	0.083	0.047	0.022	0.421	0.134
Less than high school	-0.170	0.028	-0.139	-0.090	-0.093	0.133
More than high school	0.213	-0.009	0.243	0.023	0.091	0.017
Other education	-0.075	-0.040	-0.109	0.106	0.033	0.095
Folate use before/during pregnancy	-0.094	0.121	-0.076	-0.103	0.051	0.093
Maternal Age	0.451	-0.209	0.465	-0.249	0.239	0.071
Antipsychotic use during pregnancy	0.118	0.042	0.181	0.101	-0.097	-0.164
Student	-0.226	-0.126	-0.273	0.260	0.054	-0.192
Homemaker	-0.051	0.034	-0.010	0.083	-0.093	0.177
Job seeker/other	0.071	-0.074	-0.045	-0.117	0.123	0.098
Immigrant status	0.052	0.082	-0.207	-0.051	0.365	0.072

	Before weighting	After weighting	Before weighting	After weighting	Before weighting	After weighting
	Antidepressant, ever in pregnancy vs non-exposure		Antidepressant for 3 trimesters vs non-exposure		Antidepressant for 1 trimester vs non-exposure	
Characteristics	SMD		SMD		SMD	
Contact HCP due to infertility	0.166	0.283	0.209	0.205	-0.027	0.074
Sleeping problems in pregnancy	-0.093	-0.070	-0.062	-0.175	-0.187	-0.162

Abbreviations: SMD=Standardized mean difference. SMD <0.1 indicates characteristics are satisfactorily balanced.

Table S2: Association between antidepressant use during pregnancy and postnatal SHI from sensitivity analyses

Sensitivity analysis	Fully weighted,[a,b] RR (95% CI)		
	**Antidepressants, ever in pregnancy**	**Antidepressants for 3 trimesters**	**Antidepressants for 1 trimester**
More frequent SHI vs never/hardly ever SHI			
Inclusion of 14 women with child aged 0-4 weeks	1.76 (0.78-3.95)	1.00 (0.40-2.47)	1.71 (0.57-5.18)
Excluded 5 women with psychiatric disorders other than depression/anxiety	1.90 (0.78-4.59)	0.91 (0.35-2.35)	1.89 (0.58-6.15)
Analysis with random-effect by country of residency	1.87 (0.93-3.77)	0.95 (0.29-3.07)	1.85 (0.79-4.32)
Further adjustment for use of benzodiazepines and z-hypnotics during pregnancy	1.55 (0.72-3.31)	0.89 (0.37-2.13)	1.61 (0.64-4.03)
Further adjustment for maternal age	1.92 (0.78-4.72)	0.95 (0.39-2.29)	1.89 (0.58-6.18)
Further adjustment for contact with HCP due to infertility	1.75 (0.75-4.08)	0.87 (0.36-2.10)	1.80 (0.63-5.17)
More frequent SHI vs never SHI[c]			
Inclusion of 14 women with child aged 0-4 weeks	1.55 (0.67-3.55)	0.87 (0.35-2.20)	1.39 (0.45-4.28)
Excluded 5 women with psychiatric disorders other than depression/anxiety	1.90 (0.78-4.59)	0.91 (0.35-2.35)	1.88 (0.58-6.15)
Analysis with random-effect by country of residency	1.65 (0.68-4.05)	0.82 (0.32-2.14)	1.51 (0.45-5.01)
Further adjustment for use of benzodiazepines and z-hypnotics during pregnancy	1.37 (0.63-2.98)	0.80 (0.33-1.93)	1.31 (0.51-3.38)
Further adjustment for maternal age	1.67 (0.64-4.36)	0.83 (0.34-2.00)	1.48 (0.44-4.98)
Further adjustment for contact with HCP due to infertility	1.50 (0.65-3.48)	0.78 (0.33-1.86)	1.37 (0.48-3.90)

Abbreviations: SHI=self-harm ideation; RR=Risk Ratio; CI=Confidence Interval; HCP=healthcare provider.

[a]The reference group is comprised of women non-medicated with antidepressants in pregnancy. [b]Weighted with the composite weight, constructed by multiplying the survey weight with the stabilized inverse probability of treatment weighting using maternal baseline covariates. [c]Observations reporting "hardly ever" self-harm thoughts are excluded in this comparison.

Table S3: Association between antidepressant use during pregnancy and postnatal EPDS score for items 1-9

	**n**	**Mean (95% CI)**	**Crude Mean difference (95% CI)**	**Survey weighted,[a] Mean difference (95% CI)**	**Fully weighted,[b] Mean difference (95% CI)**
Non-medicated in pregnancy	88	12.70 (11.47-13.91)	Reference	Reference	Reference
Antidepressants, ever in pregnancy	99	10.58 (9.40-11.76)	-2.11 (-3.81, -0.42)[c]	-1.65 (-3.92, 0.61)	-0.80 (-3.65, 2.05)
Antidepressants for 3 trimesters	66	10.18 (8.77-11.60)	-2.51 (-4.38, -0.65)[d]	-2.77 (-4.81, -0.74)[d]	-2.54 (-4.82, -0.27)[e]
Antidepressants for 1 trimester	22	11.73 (8.98-14.47)	-0.97 (-3.93-1.99)	1.24 (-3.91, 6.39)	1.30 (-3.06, 5.67)

Abbreviations: CI=Confidence Interval, EPDS=Edinburgh postnatal depression scale.

[a]Weighted only by the survey weight, accounting for maternal age and education.
[b]Weighted with the composite weight, constructed by multiplying the survey weight with the stabilized inverse probability of treatment weighting using maternal baseline covariates.
[c]p-value 0.015; [d]p-value 0.008-0.009; [e]p-value 0.029.
